# The effects of substrate and stacking in bilayer borophene

**DOI:** 10.1038/s41598-022-18076-0

**Published:** 2022-08-11

**Authors:** Shobair Mohammadi Mozvashi, Mojde Rezaee Givi, Meysam Bagheri Tagani

**Affiliations:** Department of Physics, University of Guilan, P. O. Box 41335-1914, Rasht, Iran

**Keywords:** Two-dimensional materials, Condensed-matter physics

## Abstract

Bilayer borophene has recently attracted much interest due to its outstanding mechanical and electronic properties. The interlayer interactions of these bilayers are reported differently in theoretical and experimental studies. Herein, we design and investigate bilayer $\beta _{12}$ borophene, by first-principles calculations. Our results show that the interlayer distance of the relaxed AA-stacked bilayer is about 2.5 Å, suggesting a van der Waals interlayer interaction. However, this is not supported by previous experiments, therefore by constraining the interlayer distance, we propose a preferred model which is close to experimental records. This preferred model has one covalent interlayer bond in every unit cell (single-pillar). Further, we argue that the preferred model is nothing but the relaxed model under a 2% compression. Additionally, we designed three substrate-supported bilayers on the Ag, Al, and Au substrates, which lead to double-pillar structures. Afterward, we investigate the AB stacking, which forms covalent bonds in the relaxed form, without the need for compression or substrate. Moreover, phonon dispersion shows that, unlike the AA stacking, the AB stacking is stable in freestanding form. Subsequently, we calculate the mechanical properties of the AA and AB stackings. The ultimate strengths of the AA and the AB stackings are 29.72 N/m at 12% strain and 23.18 N/m at 8% strain, respectively. Moreover, the calculated Young’s moduli are 419 N/m and 356 N/m for the AA and the AB stackings, respectively. These results show the superiority of bilayer borophene over bilayer $\hbox {MoS}_2$ in terms of stiffness and compliance. Our results can pave the way of future studies on bilayer borophene structures.

## Introduction

Borophene has recently attracted a surge of interest for its outstanding electronic and mechanical properties^1–6^. It is the lightest 2D material, rendering it a promising candidate for lightweight nanodevices^7–9^. Moreover, the electron deficiency of boron atoms causes complex bonding which in turn results in diverse allotropes for borophene. These different phases are defined by different arrangements of hallow hexagons (HHs) and corresponding HH concentration numbers ($\eta$ or $\nu$ in some papers). The most interested phases of borophene include $\alpha ~(\eta =1/9)$, $\beta _{12}~(\eta =1/6)$, and $\chi _3~(\eta =1/5)$^10–14^.

In addition to monolayer, bilayer borophenes have also attracted much attention. It was expected that bilayer borophene would be more stable than monolayer borophene due to the interlayer bonding^15^. To date, many theoretical and experimental works have been conducted on the subject of different bilayer borophene allotropes and their properties^15–18^. Moreover, there are still various questions to be answered. For instance, theoretical studies have suggested the interlayer distance of bilayer borophene in the range of 2.5–3 Å, suggesting a van der Waals (vdW) interaction between the layers^19–21^. However, the synthesized bilayer borophenes show a much closer interlayer distance, around 2 Å, implying relatively strong covalent bonds^22,23^. However, some theoretical studies considered some constraints to design the bilayer borophenes with similar interlayer distance to the experiment^16,24^. Formation energies and phonon dispersions prove that the constrained models are more stable than the fully relaxed models.

In this paper, by first-principles calculations, we answer why the interlayer coupling in bilayer borophene should be covalent and under what conditions this occurs. We first investigate the bilayer $\beta _{12}$ borophene without constraining the interlayer distance, or *“the relaxed model”*. Afterward, by applying the constraint of interlayer distance we reach a structure more similar to the experimental observations, phrased as *“the preferred model”*. This model, which has one covalent interlayer bond in each unit cell, is more favorable than the relaxed model. Interestingly, by applying compressive strain on the relaxed model, it undergoes a transition to the preferred model and covalent bonds form between the layers. In other words, we suggest that the preferred model is nothing but the relaxed model under compression.

The experimentally stable bilayer borophenes were synthesized on a metal substrate with negative mismatches with borophenes, which apply a compressive strain on the overlayers^22,23,25^. Otherwise, the second boron layer does not grow regularly on the first one; instead small clusters of boron form^23^. Thus, the fundamental factor for the stability of bilayer borophene and covalent interlayer bonds could be the compressive strain from the substrate. To prove this suggestion, we considered substrate-supported borophene bilayers on Al (111), Ag (111), and Au (111) surfaces and optimized the bilayer borophene on them. Our results show that the substrate-supported bilayer borophenes are more stable, with two interlayer covalent bonds in each unit cell. Our calculations show that these extra covalent bonds are due to the charge transfer from the substrate to the overlayers. This addresses well the question of why and how the bilayer borophenes can grow efficiently on the metal substrates and pave the way for future experiments. In other words, we suggest that the AA stacking of bilayer $\beta _{12}$ borophene requires a substrate with negative mismatch to be stable. On the other hand, the AB stackings of the bilayer $\beta _{12}$ borophene are stable and covalently bonded in the relaxed form. No compression nor substrate is needed. Therefore, we strongly suggest that the synthesis of an AB-stacked bilayer $\beta _{12}$ borophene is more probable than the AA-stacked one in freestanding form.

At last, we calculate and compare the mechanical properties of the AA and AB stackings. Our results show that the ultimate strength of the AA and the AB stackings are 29.72 N/m at 12% strain and 23.18 N/m at 8% strain, respectively. Moreover, Young’s moduli of the AA and the AB stackings are 419 N/m and 356 N/m, respectively, which show higher stiffness and compliance of this bilayer compared to bilayer $\hbox {MoS}_2$. Generally speaking, in this paper, we tried to exploit the most needed mechanical and structural information about the AA and AB stackings of the bilayer $\beta _{12}$ borophene, to contribute in guiding the new explorations about this subject.

## Computational details

The Spanish package solution, SIESTA^26,27^, was implemented for all the calculations, which is based on self-consistent density functional theory (DFT) and standard pseudopotentials. The exchange-correlation interactions were estimated through generalized gradient approximation (GGA), with parameterization of Perdew, Burke, and Ernzerhof (PBE)^20^. Based on the convergence of the total energy, as depicted in Supplementary Material Figs. S1 and S2, the reciprocal space was sampled by a mesh of $13\times 23\times 1$ k points in the Brillouin zone and the density mesh cut-off was set to 50 Ry. To consider the Van-der Waals interaction, the DFT-D3 correction of Grimme was implemented^22^. Moreover, a vacuum space of 20 Å was considered in the *z*-direction to prevent unwanted interactions.

The interlayer binding energy of the freestanding bilayers was calculated through:

1 $$\begin{aligned} E_b=(E_{bi}-2E_{mono})/S \end{aligned}$$

where $E_{bi}$, $E_{mono}$, and *S* are the total energy of the bilayer, total energy of each monolayer, and the area of the unit cell, respectively. The adhesion energy between the bilayer and the substrate was also calculated through:

2 $$\begin{aligned} E_{ad}=(E_{T}-E_{bi}-E_{sub})/S \end{aligned}$$

where $E_T$ is the total energy of the whole substrate-supported system and $E_{sub}$ is of the isolated substrate.

Young’s modulus is defined by:

3 $$\begin{aligned} Y_i=\frac{\partial \sigma _i}{\partial \varepsilon _i} \end{aligned}$$

where $\sigma _i$ and $\varepsilon _i$ are the stress and the strain in direction *i*. Also, $Y_{xy}$ is defined as the biaxial Young’s modulus. The stress tensor is explained in Supplementary Material Eq. (S2). The $\sigma _{11}$ and $\sigma _{22}$ directly give the stress values for strains along the armchair and zigzag directions, respectively. For the biaxial strain, the mean square values of biaxial stresses were calculated by:

4 $$\begin{aligned} \sigma _{xy}=\sqrt{\frac{\sigma _{11}^2+\sigma _{22}^2}{2}} \end{aligned}$$

Moreover, the obtained stress values were multiplied by the vacuum distance (20 Å) to get the unit of N/m. Atomic configurations and electron density map are visualized using VESTA package^28^.

## Results and discussion

### The basic model

**Figure 1:**
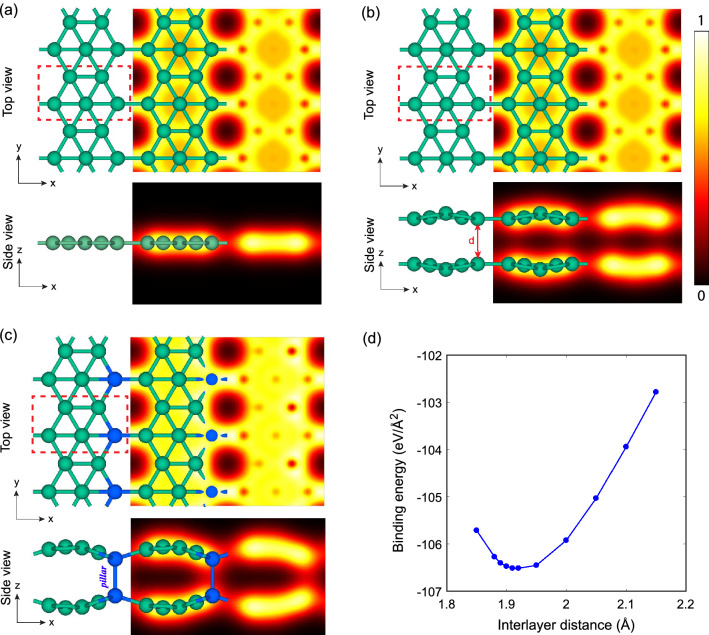
Structural configuration and electron density map of (**a**) monolayer $\beta _{12}$ borophene, (**b**) relaxed bilayer model, and (**c**) preferred bilayer model. Electron density was in the range of 0–1 e/$\text{\AA} ^3$, as shown in color bar. The unit cell, interlayer distance, and pillar bonds are also depicted. (**d**) Binding energy as a function of interlayer distance in the preferred model. The paces around the minimum were smaller to obtain more precise answer.

We start our investigations with monolayer $\beta _{12}$ borophene, which is shown in Fig. 1a. After full relaxation, a flat structure with lattice constants and average bond length of *a* = 5.15, *b* = 2.97, and *R* = 1.74 Å was obtained, which is consistent with previous theoretical and experimental records^29,30^. Subsequently, we designed and optimized an AA-stacked bilayer, as shown in Fig. 1b. The lattice constants and the average bond length are $a = 5.14$, $b = 2.98$, and $R = 1.74$ Å. The binding energy using Eq. (1) was calculated -99.5 eV/Å$^2$. The closest interlayer distance in this bilayer is $d = 2.45$ Å, which implies a van der Waals (vdW) interaction between the layers. We call this structure *“the relaxed model”*, which is in agreement with several previous works^19–21^. However many stronger theoretical and experimental works suggest a closer interlayer distance ($\sim$ 2 Å), and a covalent interlayer interaction for bilayer borophene^22–24^.

Here, two important questions arise: Why the interlayer interaction should be covalent? And why some of the theoretical works do not agree with the experiments? To address these questions, we considered a manipulated model, in which the interlayer distance was adjustable on demand. The procedure for designing and optimizing this model is described in Supplementary Material, Sect. S2. As described in Fig. 1c, we constrained this structure to have one interlayer covalent bond in each unit cell, labeled as *‘pillar’* and adjusted the interlayer distance to find the most stable state. Figure 1d shows the variation of binding energy as a function of interlayer distance in this configuration. Under these circumstances, the most favorable interlayer distance is around 1.91 Å, with a binding energy of − 106.5 eV/Å$^2$. Also, the lattice constants and the average bond length are $a = 5.06$, $b = 2.97$, and R = 1.74 Å, respectively. Interestingly, this configuration is more stable than the relaxed structure and it is more similar to the experiments, therefore, we call this structure *“the preferred model”*.

In aspects of electronic properties, the relaxed and the preferred models share similar properties. As we will discuss later, they both are metals with dominant *p* states around the Fermi level. However, as we saw in Fig. 1, the electron density maps show different interlayer interactions for these two models. There are no electrons in the interlayer space of the relaxed model, which approves the weak vdW interaction between the layers. However, in the preferred model, we can see the presence of electron density between the so-called pillar atoms, which implies covalent-like interlayer bonds. The electron localization function (ELF) and the electron difference density maps are also available in Fig. S7, approving this conclusion. All of these features suggest that the preferred model is more compatible with experimental studies^22–24^.

However, as mentioned above, the preferred model is unrealistic; for no one can hold the pillar atoms at a certain distance in real world. Then what makes this model so close to the experiment? The answer lies beneath the effects of the substrate. All the mentioned synthesized bilayer borophenes were grown on metal substrates, therefore, we should somehow take into account these effects. As we know, a substrate can influence the overlayers mechanically and electronically. The mismatch between the substrate and the overlayers can compress or stretch the latter, which affects other structural parameters including the interlayer distance. Moreover, a metal substrate, soaked in free electrons, can dope the overlayers to attract each other more strongly.

**Figure 2:**
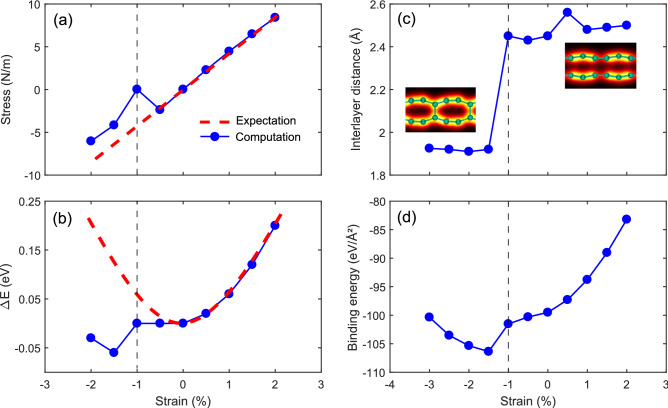
Evaluation of (**a**) stress, (**b**) total energy, (**c**) interlayer distance, and (**d**) binding energy of the relaxed model with the applied strain. The total energy was substituted from the pristine total energy ($\Delta \hbox {E} = \hbox {E} - \hbox {E}_0$). The expected harmonic behavior is shown with red dashed lines in (**a**, **b**).

We first simulate the mechanical effects of a possible substrate by applying biaxial strains on the relaxed model and observing the structural evaluation. We should keep an eye on the variation of stress and total energy with the applied strain to see if any structural phase transition takes place. In the harmonic range of a material, the stress is expected to behave linearly and the total energy to grow parabolic with compression or tension. The structural variations of the relaxed model with the applied strain are shown in Fig. 2. After a 0.5% compressive strain, the response of the stress and total energy deviates from the expected harmonic behavior, which implies a structural phase transition. Moreover, when the relaxed model is compressed around 1.5%, the binding energy and the interlayer distance drop to − 106.3 eV/Å$^2$ and 1.93 Å, respectively, which is precisely consistent with the preferred model. In other words, the relaxed model turns into the preferred model under more than 1.5% compression. This phase transition can be seen graphically in the insets of Fig. 2c. The energy curve in Fig. 2b show an energy difference of around 0.06 eV among this two models. However, the preferred model is more favorable in aspects of energy. The only controlling parameter for this transition is the applied strain. Therefore, the AA stacking requires a mismatching substrate to grow. This explains well the successful synthesis of bilayer $\beta _{12}$ sheet on Ag (111), Al (111), and Au (111) substrates, all of which have mismatches between − 1 and − 3% with borophene^22,23^. As we will further show by phonon dispersion, the AA-stacked bilayer $\beta _{12}$ is not stable in freestanding form, therefore, consideration of a proper substrate is inevitable.

### Substrate effects

**Figure 3:**
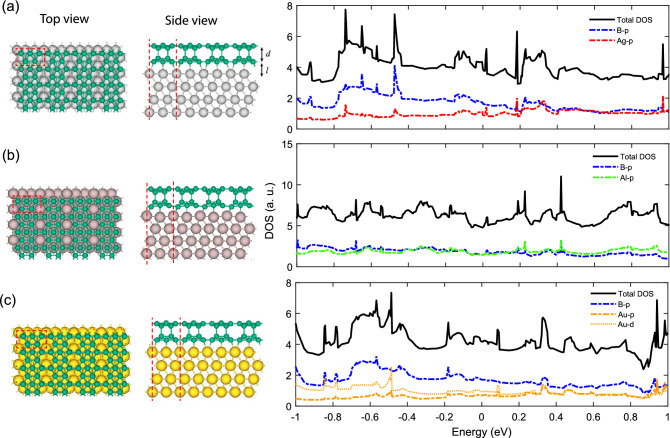
Structural configuration of (**a**) Ag-, (**b**) Al-, and (**c**) Au-supported bilayer borophenes with their partial density of states (PDOS) projected on different atomic orbitals.

To explore the electronic effects of the substrate, we designed the substrate-supported bilayer models on the Ag (111), Al (111), and Au (111) surfaces, as shown in Fig. 3. We used four layers of substrate from which the top two layers were allowed to relax and the bottom two were fixed. Besides, the borophene overlayers were allowed to fully relax. These substrates, which are among the most frequently used surfaces for borophene synthesis^31,32^, all apply compressive strains into the borophenes due to their negative mismatch of the lattice constant. The lattice constants of the optimized substrate-supported models are *a* = 5.00, *b* = 2.88 Å (Ag), *a* = 4.95, *b* = 2.86 Å (Al), and *a* = 4.99, *b* = 2.88 Å (Au), which apply compressive strains of 3%, 4%, and 3% to the bilayer, respectively. The interlayer distance (*d*) in Ag-, Al-, and Au-supported models drops to 1.90, 1.84, and 2.2 Å, which causes two covalent interlayer bonds in each unit cell to form. In other words, the compressive strain and the electrons transferred from the substrates cause the substrate-supported models to decrease the interlayer distance and make double-pillar covalently bonded bilayers. The electron doping facilities the interlayer bonding, therefore the substrate-supported models have one pillar more than our freestanding preferred model.

For a better understanding of this electron transfer, we calculated of Mulliken population of electrons among the layers. In all the substrate-supported models, the substrates donate and the overlayers accept electrons. In the Ag-supported model, the substrate averagely donates 7 $\times 10^{17}$ e/m$^2$ to the overlayer, where the lower and the upper layers averagely accept their shares as 3$\times 10^{17}$ and 4$\times 10^{17}$ e/m$^2$, respectively. These results support the previous theoretical study of borophene bilayer on Ag (111) substrate^15^. In the Al-supported model the substrate donates an average of $3\times 10^{17}\hbox { e/m}^2$, from which the lower and the upper layers accept $1\times 10^{17}$ and $2\times 10^{17}\hbox { e/m}^2$, respectively. Moreover, in the Au-supported model the substrate donates around $12\ \times 10^{17}\hbox { e/m}^2$ from which the upper and the lower borophenes accept $5\times 10^{17}$ and $7\times 10^{17}\hbox { e/m}^2$, respectively.

The injection of electron from the substrate compensates the electron deficiency of the borophene, providing a good condition for the two layers to make covalent bonds. The upper layer only makes bonds with the lower layer whereas the lower layer pays more electrons to make bonds with both the substrate and the upper layer, therefore the Mulliken population of the upper layer is higher. This also suggests for likelihood of production of more than two-layer structures, opening a way to obtain the bulk layered boron. We also calculated the adhesion energy between the substrates and the overlayer, which are − 0.18, − 0.15, and − 0.22 eV/Å$^2$ for Ag-, Al-, and Au-supported models, respectively. These values show more adhesion in comparison with the $\eta _{1/12}$ borophene on the Ag (111) substrate (− 0.11 eV/Å$^2$)^15^.

Figure 3 also shows the orbital projected partial density of states (PDOS) of the substrate-supported bilayers. In all three models, more or less, the most dominant states around the Fermi level are *p* orbitals of the B and substrate (Ag, Al, or Au) atoms. In Au-supported system, Au-*d* orbital also have important contribution. Other insignificant states were excluded from the plot for more clarity. In Ag-supported model, B-*p* states are the most dominant in the valance band, but in the conduction band, the B-*p* and Ag-*p* states make an orbital hybridization, where this two states have similar contribution. In Al-supported model, B-*p* and Al-*p* orbitals strongly hybridize together in both valance and conduction bands in the considered range, which is a signature of strong covalent bonds. However, in Au-supported model, the dominance of valance and conduction bands are with the B-*p* states and no apparent hybridization takes place between the orbitals of B and Au atoms. It is worth mentioning that, in the valance band, the Au-*d* are more dominant than Au-*p* orbitals, while in the conduction band they hybridize only with each other. It is clear that the level of orbital hybridization is correlated to the substrate-overlayer distance (*l*), as shown in Table 1. This table also shows a better comparison of the proposed substrate-supported models in other aspects.

**Table 1:** Structural properties of the substrate-supported bilayer $\beta _{12}$ borophenes: lattice constants (*a* and *b*), interlayer distance (*d*), substrate-overlayer distance (*l*), substrate adhesion energy ($E_{ad}$), number of covalent bonds per unit cell ($n_B$), and density of electrons donated from the substrate ($\rho _d$).

Substrate	*a* (Å)	*b* (Å)	*d* (Å)	*l* (Å)	$E_{ad}$ (eV/Å$^2$)	$n_B$	$\rho _d$ ($10^{17}\hbox { e/m}^2$)
Ag (111)	5.00	2.88	1.90	2.00	$-$ 0.18	2	7
Al (111)	4.95	2.86	1.84	1.68	$-$ 0.15	2	3
Au (111)	4.99	2.88	2.20	2.23	$-$ 0.22	2	12

### Stacking effects

**Figure 4:**
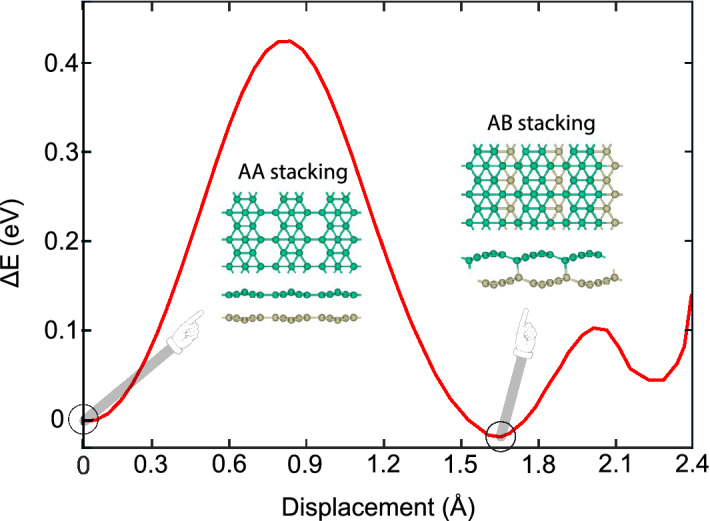
Variation of energy as a function of displacement along the armchair direction. The energy of AA stacking was set to zero. The AA and AB stackings are shown in the insets.

Up to this point, we only concentrated on the AA stacking of $\beta _{12}$ bilayer borophene as the basic configuration. To take into account the effects of layer displacement, we moved the upper layer regarding the lower one along the armchair direction. The displacement along the zigzag direction is not favored because the dangling bonds cause a rapid instability. The variation of energy with armchair displacement is shown in Fig. 4. The energy ascends to a maximum of 0.42 eV under a displacement of around 0.8 Å. Then it descends down to the global minimum of − 0.04 eV under a displacement of around 1.7 Å. After that, in spite of a non-significant relative minimum, the energy ascends and descends in a repetitive trend until the bilayer turns into the AA stacking again. As shown in the inset, the global minimum takes place for the AB stacking, where the 6-folded B atoms of the upper layer are placed above the hexagon holes of the lower one. After full relaxation, this structure has the lattice constants of *a* = 5.03 Å, *b* = 2.97 Å, the average bond length of *R* = 1.76 Å, and binding energy of − 114.43 eV/Å$^2$.

**Figure 5:**
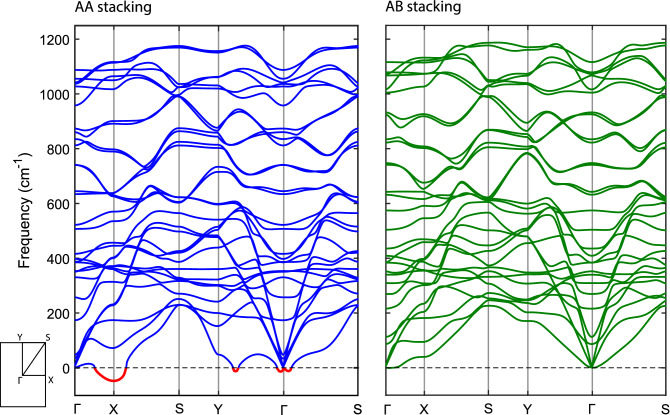
Phonon dispersion of bilayer $\beta _{12}$ borophene with AA (left) and AB (right) stackings. The Brillouin zone is also depicted in the inset.

**Figure 6:**
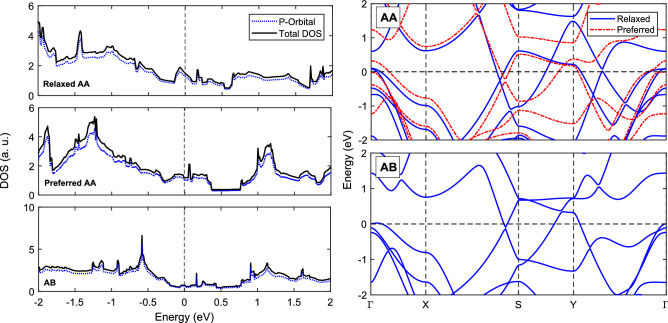
Density of states (DOS) and band structures of the AA and AB stacked $\beta _{12}$ bilayer borophene models.

Interestingly, unlike the AA stacking, the AB stacking has a covalent interlayer interaction in the relaxed form with $d = 2$ Å. Thus, the AB stacking does not need the support of a substrate to have covalent interlayer bonds. The presence of covalent bonds among the layers is expected to improve the stability of the bilayer. To see these effects, we calculated phonon dispersions of both relaxed AA and AB stackings and compared them in Fig. 5. The AA stacking has several negative modes with values of tens of cm$^{-1}$, which are signatures of dynamical instability. Interestingly, in the AB stacking phonon bands, no imaginary modes are seen, which approves its high stability. This suggests that, in a potential experiment, the bilayer $\beta _{12}$ borophene is very likely to grow with AB stacking in freestanding form. For a better comparison between the AA and AB stackings, please pay attention to Table 2.

**Table 2:** Comparison between different models in the freestanding form: lattice constants (*a* and *b*), interlayer distance (*d*), binding energy ($E_b$) and number of interlayer bonds in a unit cell ($n_b$).

Model	*a* (Å)	*b* (Å)	*d* (Å)	$E_b$ (eV/Å$^2$)	$n_b$
**AA**					
Relaxed	5.14	2.98	2.45	− 99.53	0
Preferred	5.06	2.97	1.91	− 106.52	1
AB	5.03	2.97	2.00	− 114.43	1

The band structures and partial density of states (PDOS) in Fig. 6 shows that, despite the differences in the interlayer bonding, the AA and AB stackings share most of the electronic properties including metallicity and orbital composition in density of states. The three models (relaxed AA, preferred AA, and AB) are metals with domination of p states near the Fermi level. For the importance of the mechanical properties for applications of 2D materials, in the following section, we report and compare the mechanical properties of the bilayer $\beta _{12}$ borophene with the AA and AB stackings.

**Figure 7:**
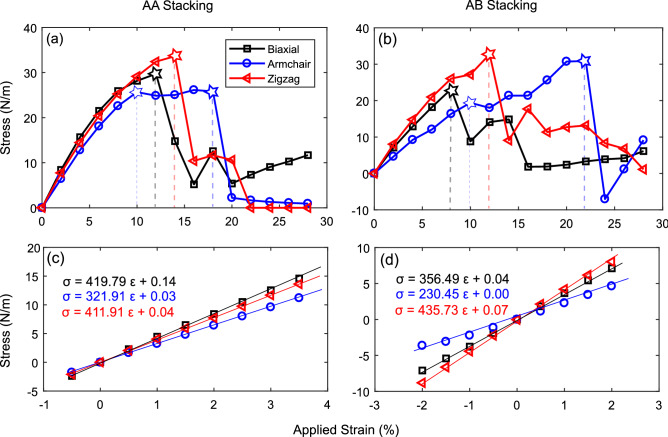
Mechanical properties of $\beta _{12}$ borophene with AA and AB stacking. (**a, b**) Long-range stress-strain curves used to find the critical strains and ultimate strengths. (**c, d**) Short-range stress-strain curves in the harmonic range used to calculate Young’s moduli.

### Mechanical properties

Knowing the mechanical properties of a material is very vital for its applications in nanodevices. Here, we report critical strains, ultimate strengths and Young’s moduli for bilayer $\beta _{12}$ borophene with AA and AB stackings. Here, the relaxed structures were used and no constraints were applied on the interlayer distances. We first applied tensile strain in the range of 0–30% to evaluate the mechanical strength of the structures. As shown in Fig. 7a, with biaxial strain in the range of 0–12%, the stress of AA stacking rises to 29.72 N/m. Afterward, it suddenly drops to lower values. This gives us the critical strain and the ultimate strength under biaxial tension. For the zigzag direction, a critical strain of 14% gives the ultimate strength of 33.63 N/m, mildly higher than the biaxial ones. In the case of the armchair direction, we have two critical strains of 10% and 18%, which give closely equal yield and ultimate strengths^33,34^ of around 26 N/m.

A similar investigation was done for the AB stacking, shown in Fig. 7b. The critical biaxial strain of 8% gives an ultimate strength of 23.18 N/m. Moreover, the zigzag direction comes with critical strain and ultimate strength of 12% and 32.74 N/m, respectively. Again, the armchair direction has two critical strains of 10% and 22%, with yield and ultimate strengths of 19.41 N/m and 30.89 N/m, respectively. Regardless of the stacking, we can see that $\beta _{12}$ bilayer has a more complicated mechanical behavior in the armchair direction. This might be due to the more complex bonding characteristics in this direction.

Defined by Eq. (3), Young’s modulus is the gradient of the stress-strain relation in the harmonic range. The harmonic range for the AA and AB stacking is 0–4% and − 2 to + 2%, respectively. The unconventional harmonic range of the AA stacking is due to the structural phase transition at − 0.5%, which was discussed before. As shown in Fig. 7c,d, Young’s moduli of the AA stacking are 420, 322, and 412 N/m for biaxial, armchair, and zigzag strains. In a similar order, the AB stacking comes with 356, 230, and 436 N/m, respectively. Thus, the AB stacking is softer along the biaxial and armchair directions, but mildly stiffer along the zigzag direction. We compared the obtained mechanical properties with graphene, BN, and $\hbox {MoS}_2$ bilayers in Table 3. Overall, we suggest that the stiffness and compliance of $\beta _{12}$ bilayer borophene is higher than $\hbox {MoS}_2$, but lower than graphene and BN.

**Table 3:** Mechanical properties of AA and AB-stacked bilayer borophene, compared with other 2D bilayers including graphene, BN, and $\hbox {MoS}_2$: Young’s moduli ($Y_{ii}$), critical strain ($\varepsilon$*), and ultimate strength ($\sigma$*).

	Yx (N/m)	Yy (N/m)	Yxy (N/m)	$\varepsilon$* (%)	$\sigma$* (N/m)
$\beta _{12}$ bilayer (AA)	322	412	420	12	29.7
$\beta _{12}$ bilayer (AB)	356	230	436	8	23.2
Bilayer graphene^35^	–	–	620	11	72.1
Bilayer BN^35^	–	–	560	12	42.4
Bilayer $\hbox {MoS}_2$^36^	–	–	260	10	28.0

## Conclusion

In summary, by first-principles calculations, we investigated the bilayer $\beta _{12}$ borophenes with different structures. We suggest that the interlayer bonding plays an important role in the stability of the bilayer. The AA stacking cannot make covalent interlayer bonds spontaneously, therefore it cannot grow in freestanding form. It requires a metal substrate such as Ag (111), Al (111), and Au (111) to be stable. These substrates, by applying compressive strain and doping electrons, help the two boron layers to attract each other more closely and make interlayer bonds. However, the AB stacking has covalent interlayer bonds which makes it stable in freestanding form. This is approved by phonon dispersion analysis. We also calculated the mechanical properties of the AA and AB stackings, which show higher stiffness and compliance of bilayer $\beta _{12}$ borophene than bilayer $\hbox {MoS}_2$. This results can give a positive contribution for future explorations about bilayer borophene structures. Moreover, for its light atomic mass, high mechanical compliance, and metallic nature, a wide range of applications can be inspired for bilayer $\beta _{12}$ borophene, including energy storage, bio-sensing, and electrodes.

## Supplementary Information

Supplementary Information.

## Data Availability

The datasets used and/or analyzed during the current study available from the corresponding author on reasonable request.
