# Supplementary material for: The effects of substrate and stacking in bilayer borophene
Source: Sci Rep. 2022 Aug 11;12:13661. doi: 10.1038/s41598-022-18076-0 (PMC9372144; doi:10.1038/s41598-022-18076-0)
Supplement: Supplementary file 1 — Supplementary Information. [file 41598_2022_18076_MOESM1_ESM.pdf]

Supplementary Material for:

The effects of substrate and stacking in bilayer borophene

Shobair Mohammadi Mozvashi[1], Mojde Rezaee Kivi[1], Meysam Bagheri Tagani[1]

[1]Computational Nanophysics Laboratory (CNL), Department of Physics, University of Guilan, P. O. Box 41335-1914, Rasht, Iran.

Sec. S1: Computational details

Before the beginning of the investigations, the proper k-points and energy cut-off should be selected. We did so by finding the k-point and energy cut-off at which the total energy of a single layer beta-12 borophene is converged to a certain number. We first found the converging k-point at a 50 Ry cut-off. The converging k-point was found to be 13x21. Afterward, we repeated the converging process at 13×21×1 k-points with different cut-offs. The 50 Ry cut-off is then confirmed. Figure S1 and Figure S2 show the detailed results.

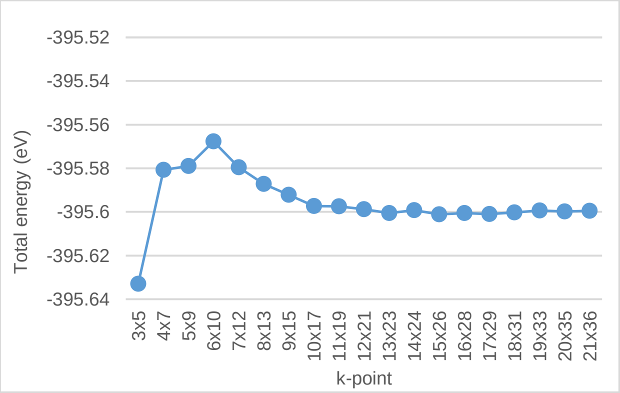

Figure S1. Convergence of total energy with different k-points.

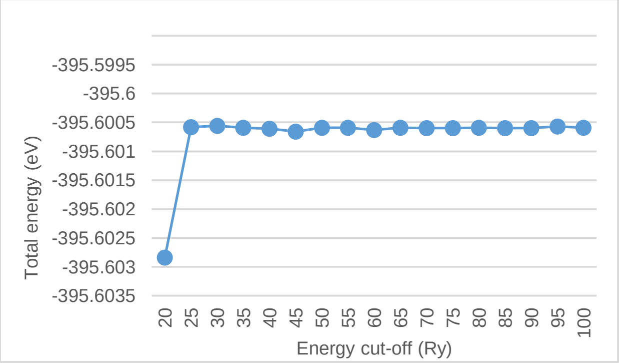

Figure S2. Convergence of total energy with energy cut-off.

The stress tensor is defined by:

$$Stress\ Tensor = \begin{pmatrix} S_{11} & S_{12} & S_{13} \\ S_{21} & S_{22} & S_{23} \\ S_{31} & S_{32} & S_{33} \end{pmatrix} eV/Å^3 \qquad (S1)$$

Sec. S2: Structural configurations

The relaxed model is the one that we optimized completely with no constraints on the interlayer distance. As you can see in Figure S3, a high interlayer distance is predicted for this model.

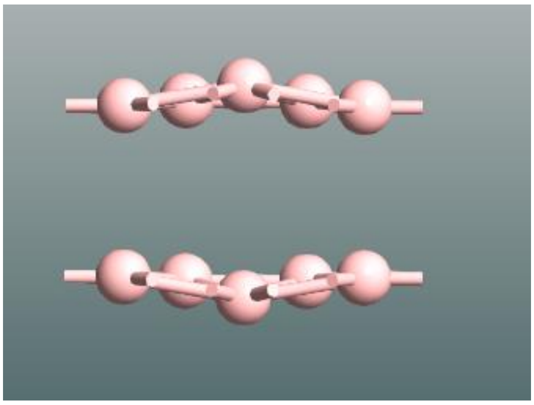

Figure S3. The structural configuration of vdW-bonded AA-stacked bilayer borophene.

In the preferred model, we took constraints to keep the interlayer distance at the covalent-like bonding limit. As we can see in Figure S4, there are three atomic sites in a β-12 borophene unit cell. Each site A, B, and C boron atom has four, five, and six covalent bonds with other boron atoms, respectively. Therefore, the Site **A** atoms are good candidates for forming covalent bonds with the other borophene layer. These interlayer covalent bonds are called ‘*pillars*’.

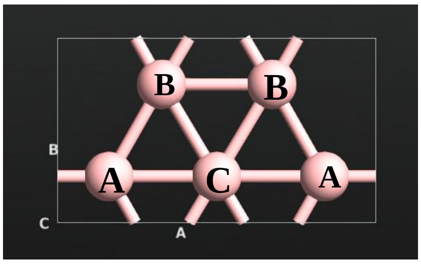

Figure S4. Three atomic sites in β-12 borophene

Two states can be considered: single pillar and double pillar as shown in Figure S5. The two atoms forming a pillar are regarded as a ‘rigid body’, therefore the interlayer distance can be

adjusted on-demand. By performing the binding energy analysis, we concluded that the single pillar with interlayer distance of 1.91 Å is the preferred state, as shown in Figure S6. This state is also the same as the model used in Li *et al* (2019) [1].

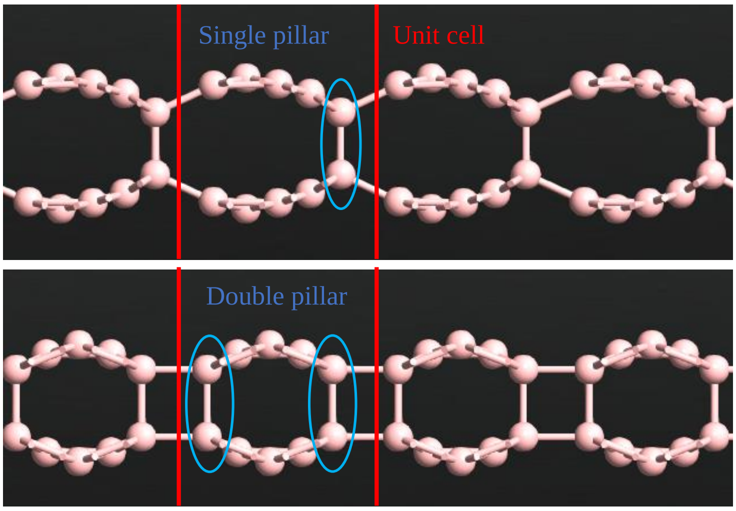

Figure S5. Single pillar (upper row) and double pillar (lower row) models.

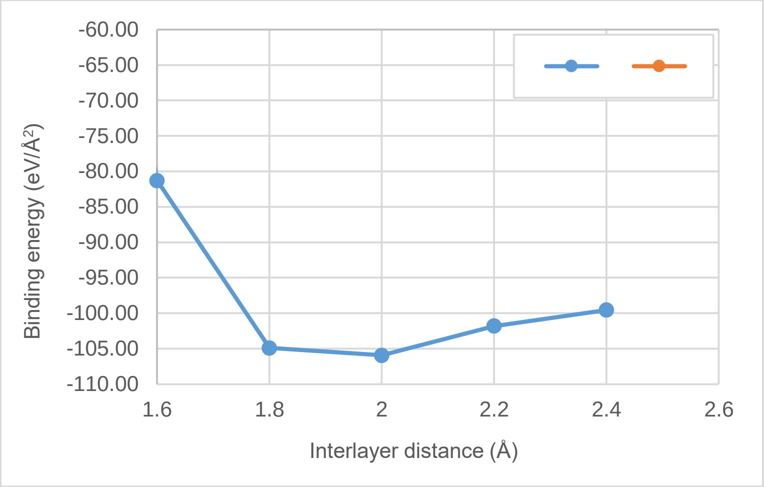

Figure S6. The binding energy of the single and double pillar CV model with interlayer distance.

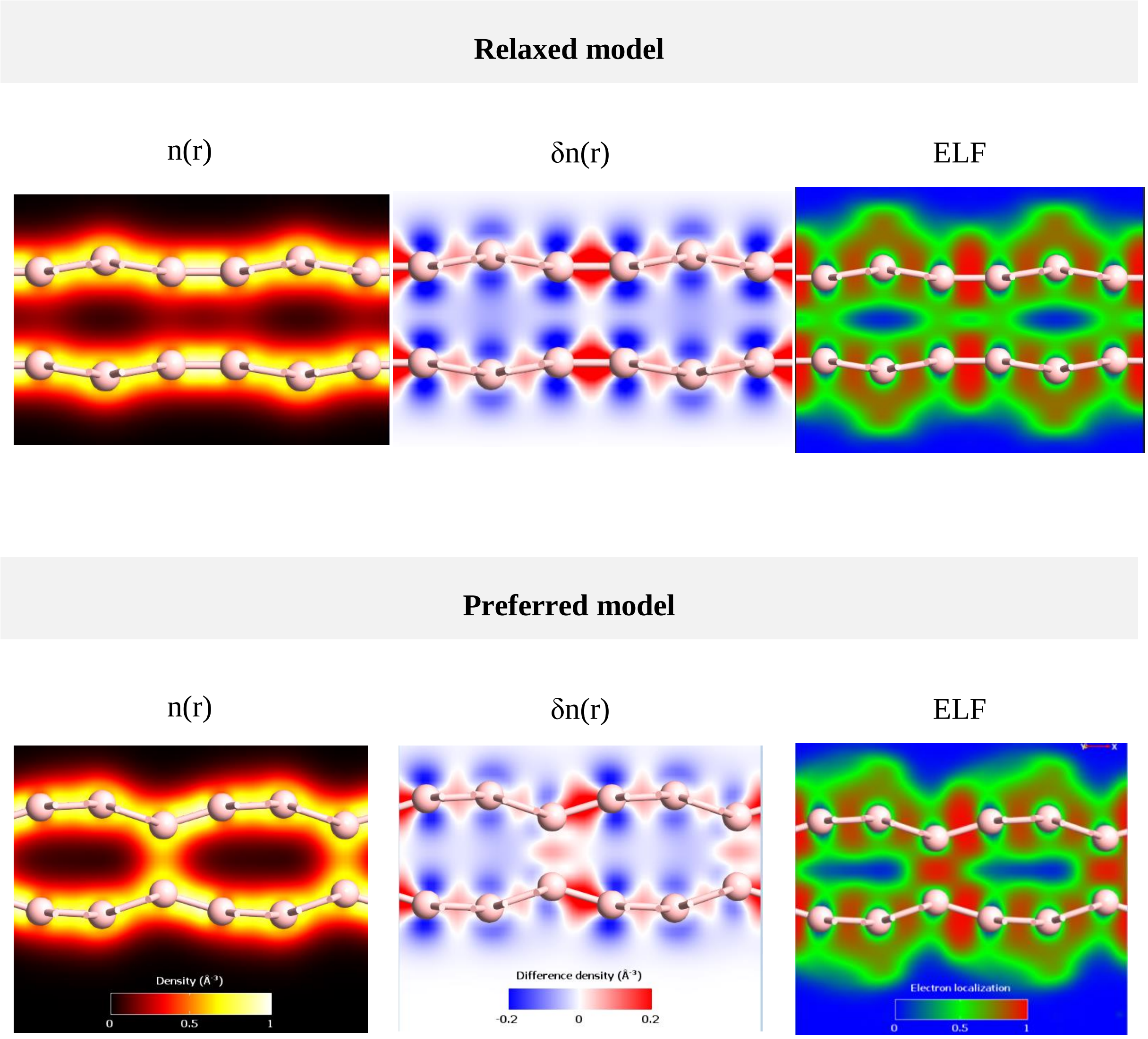

Figure S7. Electron density, n(r), electron difference density, δn(r), and electron localization function, ELF, of the relaxed and the preferred models.

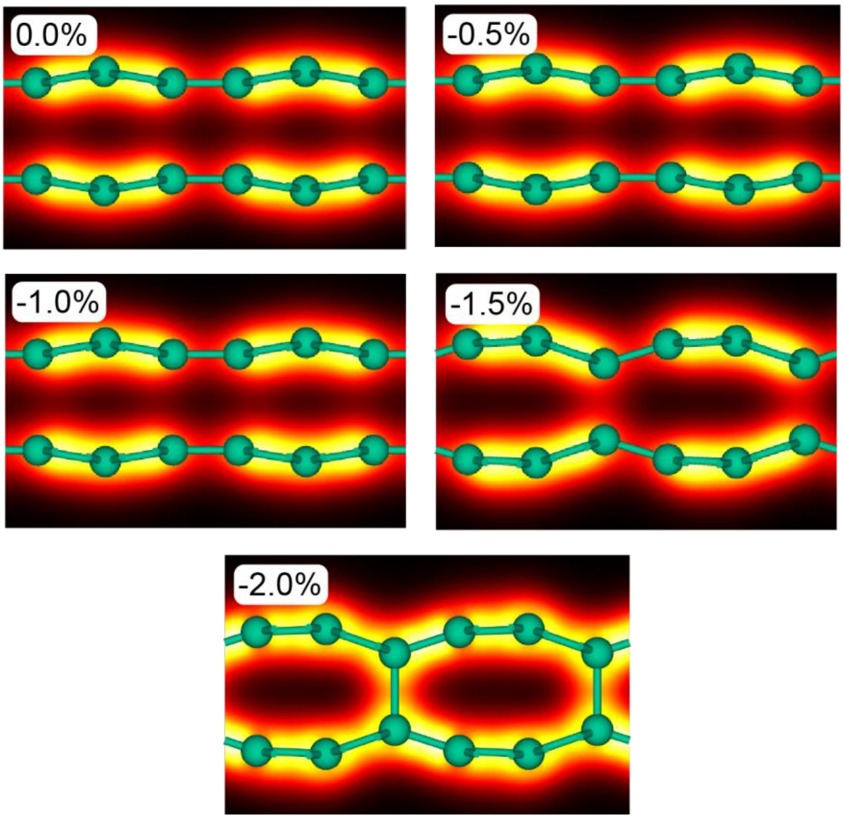

Figure S8. Structural evaluation of the relaxed bilayer borophene with the applied strains, along with the electron density maps.

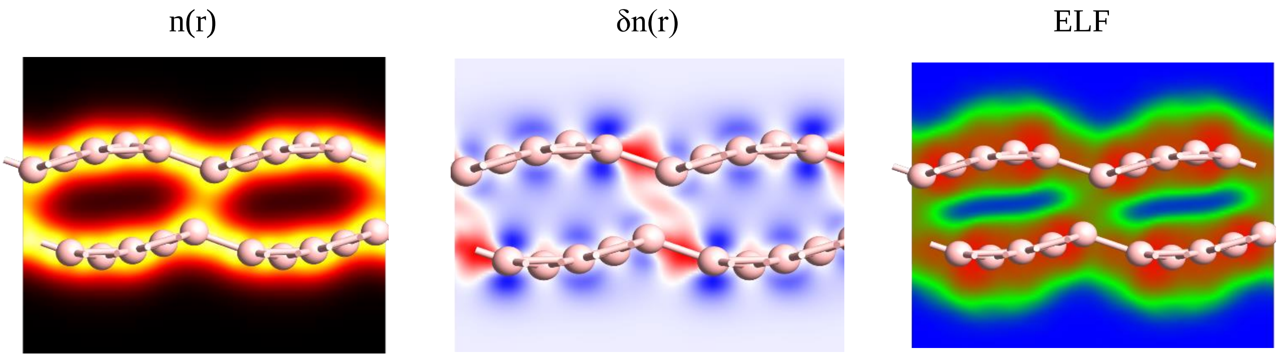

Figure S9. Electron density, n(r), electron difference density, δn(r), and electron localization function, ELF, of the AB stacking.

References

[1] D. Li *et al.*, "From two-to three-dimensional van der Waals layered structures of boron crystals: an ab initio study," *ACS Omega*, vol. 4, no. 5, pp. 8015-8021, 2019.
